# Effect of illicit direct to consumer advertising on use of etanercept, mometasone, and tegaserod in Canada: controlled longitudinal study

**DOI:** 10.1136/bmj.a1055

**Published:** 2008-09-02

**Authors:** Michael R Law, Sumit R Majumdar, Stephen B Soumerai

**Affiliations:** 1 Department of Ambulatory Care and Prevention, Harvard Medical School and Harvard Pilgrim Health Care, 133 Brookline Avenue, 6th floor, Boston, MA 02215, USA; 2 Division of General Internal Medicine, Department of Medicine, 2E3.07 Walter Mackenzie Health Sciences Centre, University of Alberta Hospital, Edmonton, Alberta T6G 2B7, Canada

## Abstract

**Objective** To assess the impact of direct to consumer advertising of prescription drugs in the United States on Canadian prescribing rates for three heavily marketed drugs—etanercept, mometasone, and tegaserod.

**Design** Controlled quasi-experimental study using interrupted time series analysis.

**Population** Representative sample of 2700 Canadian pharmacies and prescription data from 50 US Medicaid programmes.

**Main outcome measures** Differences in number of filled prescriptions per 10 000 population per month between English speaking and French speaking (control) Canadian provinces before and after the start of direct to consumer advertising in the United States.

**Results** Spending on direct to consumer advertising for study drugs ranged from $194m to $314m (£104m-£169m; €131m-€212m) over the study period. Prescription rates for etanercept and mometasone did not increase in English speaking provinces relative to French speaking controls after the start of direct to consumer advertising. In contrast, tegaserod prescriptions increased 42% (0.56 prescriptions/10 000 residents, 95% confidence interval 0.37 to 0.76) in English speaking provinces immediately after the start of US direct to consumer advertising. Uncontrolled analysis of US Medicaid data showed a larger 56% increase in tegaserod prescriptions. However, this increase did not persist over time in either country, despite continued advertising.

**Conclusions** Exposure to US direct to consumer advertising transiently influenced both Canadian and US prescribing rates for tegaserod, a drug later withdrawn owing to safety concerns. The impact of direct to consumer advertising on drug use seems to be highly variable and probably depends on the characteristics of the advertised drug, the level of exposure to direct to consumer advertising, and the cultural context.

## Introduction

Direct to consumer advertising is a major component of drug promotion in the United States; manufacturers spent an estimated $4.24bn (£2.28bn; €2.88bn) in 2005—a 330% increase since 1996.^1^ The merits of direct to consumer advertising have been extensively debated, which has led to differing regulations across countries.^2^ ^3^ Regulatory disputes continue worldwide, with ongoing debate about the introduction of direct to consumer advertising in the European Union and Canada; at the same time, the US Senate has recently considered legislation prohibiting such advertising during the first two years after the release of a new drug.^4^ ^5^ ^6^ Although the debate includes a broad range of concerns, many assertions assume that direct to consumer advertising increases the use of particular types of drugs. For example, proponents argue that it increases use of effective treatments for undertreated conditions, such as depression.^2^ Opponents, however, suggest that it drives up demand for newer drugs with higher costs, marginal benefits, and unknown safety profiles.^3^

Both sides of the argument assume that direct to consumer advertising increases use. However, the effectiveness of drug advertising campaigns is unclear and no extant studies use a concurrent control group and quantify the impact on use of marketed drugs.^7^ ^8^ Previous uncontrolled longitudinal studies have found that expenditure on direct to consumer advertising was associated with higher sales of antidepressants, proton pump inhibitors, antihistamines, and nasal sprays but non-significant or very small association with sales of statins and cyclo-oxygenase-2 selective non-steroidal anti-inflammatory drugs.^9^ ^10^ ^11^ ^12^ How these associations might be confounded by selection bias is unclear from these previous studies. For example, drugs with a larger pool of potential users or that are more innovative are more likely to be promoted through both direct to consumer advertising and physician directed campaigns using detailing, journal advertisements, and free samples.^13^ Moreover, previous studies have not controlled for pre-advertising trends in use or evaluated comparable markets that are unexposed to such advertising.

In the absence of firm evidence describing the effect of direct to consumer advertising on use of prescription drugs, policy makers in the United States and New Zealand have permitted it whereas their counterparts in Europe, Canada, and Australia have prohibited it. The extent of benefits or harms attributable to direct to consumer advertising will be directly proportional to how effectively it increases use of particular advertised drugs and at what cost. We studied the impact of US direct to consumer advertising campaigns on Canadian prescribing rates for three heavily marketed drugs by using a controlled longitudinal study design. Because Canadians are regularly exposed to “illicit” English language direct to consumer advertising from the United States, we hypothesised that these campaigns would increase use of the marketed drugs in English speaking Canadian provinces. For any campaigns associated with increased use in our Canadian analysis, we examined US Medicaid data without a control group to investigate whether the effects were greater with increased exposure to direct to consumer advertising.

## Methods

### Study setting

Examination of US data alone to delineate the impact of direct to consumer advertising is limited by two factors. Firstly, near universal exposure to advertising makes it almost impossible to find a comparable unexposed control group within the United States.^14^ Secondly, manufacturers start many direct to consumer advertising campaigns shortly after the launch of a drug—precisely when detailing to physicians and coverage in the medical literature are likely to be at their highest. We sought to limit these threats to validity by examining the impact of US direct to consumer advertising campaigns on Canadian patterns of drug use in provinces with and without substantial exposure to such advertising—that is, in predominantly English speaking provinces compared with predominantly French speaking Quebec. For drugs for which we found an impact on Canadian prescribing rates, we used data from nationwide US Medicaid programmes to assess whether a dose-response relation might exist between greater exposure to direct to consumer advertising in the United States and more marked increases in drug use.

Although Canada prohibits direct to consumer advertising that includes both a brand name and indications, substantial cross border exposure to US advertising occurs through cable and satellite television, radio, print media, and internet advertising.^15^ Statistics Canada estimates that around 30% of television watched by English speaking Canadians is foreign sourced, most of which is probably US cable and satellite stations.^16^ Previous Canadian survey work suggested that more than 85% of English speaking patients had seen drug advertisements in the previous year and half had seen advertisements for six or more different products.^15^ Moreover, their primary care physicians filled nearly three quarters of patients’ requests for specific drugs.^15^ Thus, English speaking Canadians are regularly exposed to considerable amounts of US advertising and have the means to obtain advertised drugs.

### Data sources

Our primary analysis used monthly drug use data from the nationally representative CompuScript audit from IMS Health Canada, an independent health information company, from January 2002 to December 2006. This audit uses a panel of approximately 2700 pharmacies (roughly 34% of all community pharmacies in Canada) to estimate total Canadian use of each drug. The major outcome of interest was the number of dispensed prescriptions of each drug per 10 000 residents per month. To calculate these rates, we used population estimates from Statistics Canada.^17^ We also obtained IMS Health Canada data estimating Canadian expenditure on detailing and distribution of free samples for the study drugs, to assess whether other marketing increased coincidently with US direct to consumer advertising. We found no evidence of such changes. Our analysis in the United States used quarterly data from 50 US Medicaid programmes.^18^ Using state level enrolment numbers, we calculated dispensed prescription rates per 10 000 Medicaid enrolees per quarter.^19^ These data provide estimates up to the end of 2005, when many patients were transferred to the new Medicare Drug Benefit.

The start month and total spending on US direct to consumer advertising campaigns came from TNS Media Intelligence. The dataset tracks advertising and estimates expenditure across several media, including television, radio, and print media, and has been used in previous research on direct to consumer advertising.^9^ We also searched the Vanderbilt Television news archive to ascertain when particular drugs were advertised during major US national news broadcasts.^20^ Finally, we assessed whether manufacturers aired television advertising in Canada mentioning a brand name by reviewing the databases of Eloda, an independent company that provides monitoring and verification services for North American advertising.

### Study drugs

Differences exist between the United States and Canada in terms of availability and approval dates for drugs.^21^ Consequently, we sought out drugs that were included in US marketing campaigns started between January 2003 and December 2005; not advertised on Canadian television with a brand name; and approved for use in Canada before US advertising, to allow estimation of the marginal effect of direct to consumer advertising on prescribing.

On the basis of these characteristics, we identified three study drugs. The first eligible drug was etanercept (Enbrel), a biological agent approved in Canada for the treatment of symptom refractory rheumatoid arthritis. Direct to consumer advertising for etanercept started in January 2003, and US network news advertising started in March 2003.^20^ The second eligible drug was mometasone (Nasonex), an inhaled nasal steroid spray for symptoms of allergy. Direct to consumer advertising for mometasone started in December 2004, and the Vanderbilt database showed extensive US news advertising starting the same month.^20^ Thirdly, tegaserod (Zelnorm) is a serotonin receptor agonist approved for the treatment of constipation predominant irritable bowel syndrome in women. When released, it was the only drug approved specifically for this indication in Canada. Although direct to consumer advertising began in February 2003, tegaserod’s most influential and major campaign first aired in August 2003 and featured memorable written messages such as “I feel better” on actresses’ stomachs.^6^ This later campaign was considered very successful from a marketing perspective, and even won major advertising industry awards, before the drug was withdrawn in both Canada and the United States owing to concern about cardiac side effects.^6^ ^22^ The Vanderbilt database indicates that US newscast advertising for tegaserod occurred in 1-12 September 2003 and subsequently in March 2004.^20^

### Analysis

We used regional differences in exposure to investigate the impact of direct to consumer advertising. As all US advertising was in English, we hypothesised that changes in prescribing in Canada would be concentrated in predominantly English speaking provinces. Although French speaking Canadians watch a similar amount of television, they view much less foreign sourced television, estimated at less than 5% of all viewing.^16^ Consequently, we analysed the difference in prescribing rates between predominantly English speaking provinces (n=8) and Quebec, where French is the mother tongue for more than 80% of the population.^23^ Quebec is also attractive as a control as it has one of the least restrictive public drug formularies in Canada but has comparable universal health insurance coverage, age, sex, and income profiles to the other provinces.^24^ ^25^

We used interrupted time series analysis, one of the strongest quasi-experimental designs available, to examine longitudinal changes in Canadian prescribing rates.^26^ Firstly, we calculated the difference in the prescribing rate per 10 000 population by subtracting the rate in French speaking provinces from that in English speaking provinces. We then fitted time series models to test whether a statistically significant change occurred in the level or trend of the difference after the start of US advertising or US national network news advertising, controlling for the pre-direct to consumer advertising level and trend. This method simultaneously controlled for any pre-advertising differences in the absolute level of prescribing between the provinces as well as any differences in pre-advertising temporal trends related to changes in the rates of prescribing between provinces. We also did a sensitivity analysis using the ratio of English and French prescribing rates instead of the difference. The results and interpretation of this analysis (not shown) were consistent with those shown below. For drugs that showed any significant impact of direct to consumer advertising in Canada, we did a sensitivity analysis using data from US Medicaid programmes. We also did this with an interrupted time series analysis but without an “unexposed” US control group. Although this method is uncontrolled compared with the Canadian analyses, it still controlled for pre-direct to consumer advertising trends in drug use. We used a generalised least squares model allowing for a first order autoregressive correlation between consecutive months or quarters and excluded the advertising start month in Canada. We validated our use of this autocorrelation structure by using likelihood ratio tests. Moreover, alternative models with no or longer autocorrelation structures led to results with very similar estimates and identical interpretations.

## Results

Table 1 describes the US advertising campaigns and Canadian approval dates for the three study drugs. All three drugs had large direct to consumer advertising expenditures, ranging from US$194 million to $314 million during the study period. Pre-advertising trends in use for each of the study drugs were generally comparable between English speaking and French speaking provinces (figs 1, 2 and 3). We found that US direct to consumer advertising led to increased Canadian prescribing rates for only one of the three drugs, tegaserod.

**Table tbl1:** US approval and advertising dates and Canadian approval dates for study drugs

Drug		United States				Canada
Generic name	Brand name	Approval	Advertising start	DTCA spending to 2006 ($m)		Approval
Etanercept	Enbrel	November 1998	January 2003	$194		December 2000
Mometasone	Nasonex	October 1997	December 2004	$235		July 1998
Tegaserod	Zelnorm	July 2002	February 2003	$314		March 2002

DTCA=direct to consumer advertising.

Start dates and US advertising values are from TNS Media Intelligence. Data include spending on network and cable television, magazine, newspaper, radio, and billboard advertising.

**Figure fig1:**
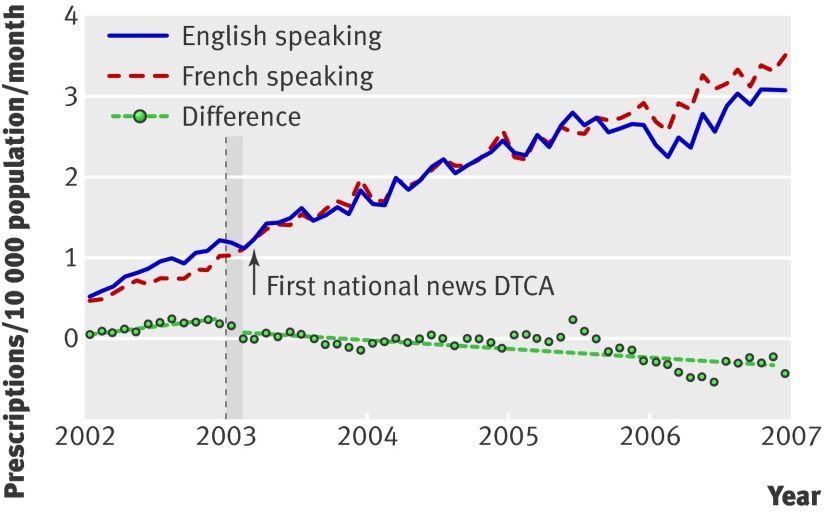
**Fig 1** Number of etanercept prescriptions per 10 000 population per month in Canadian provinces that are predominantly English speaking (n=8) or French speaking (n=1). Vertical line indicates start of US advertising in January 2003. Difference between rates shown at bottom of chart; fitted trend line shows predicted differences from interrupted time series regression. DTCA=direct to consumer advertising

**Figure fig2:**
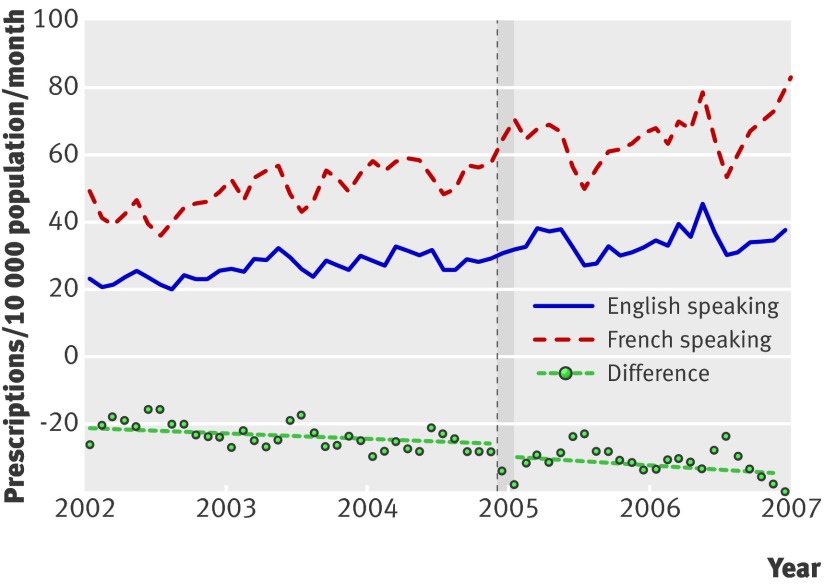
**Fig 2** Number of mometasone prescriptions per 10 000 population per month in Canadian provinces that are predominantly English speaking (n=8) or French speaking (n=1). Vertical line indicates start of US advertising in December 2004. Difference between rates shown at bottom of chart; fitted trend line shows predicted differences from interrupted time series regression

**Figure fig3:**
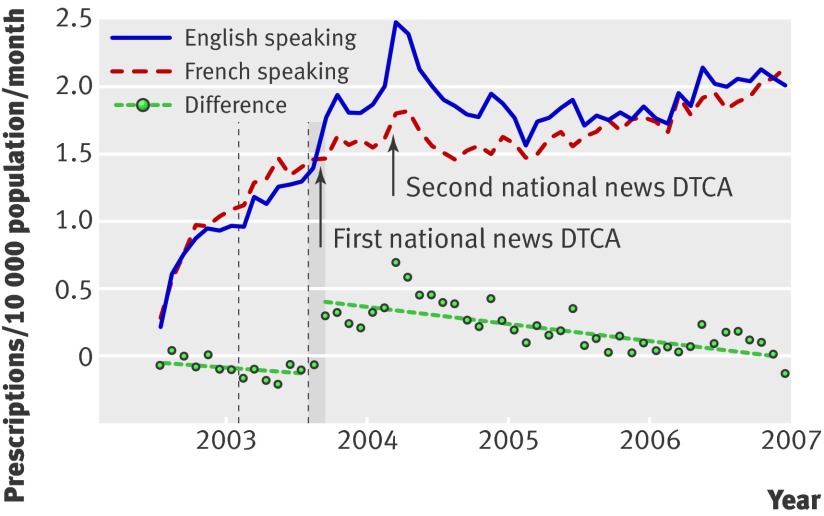
**Fig 3** Number of tegaserod prescriptions per 10 000 population per month in Canadian provinces that are predominantly English speaking (n=8) or French speaking (n=1). Vertical lines indicate start of US advertising in February 2003 and start of new TV advertising campaign in August 2003. Difference between rates shown at bottom of chart; fitted trend line shows predicted differences from interrupted time series regression. DTCA=direct to consumer advertising

### Etanercept

Figure 1 shows the times series of monthly prescribing rates of etanercept in Canada, which were very similar in both language regions. We found that advertising had no statistically significant impact on the level or trend of differences in prescribing rate between English speaking and French speaking provinces (level change −0.18 prescriptions per 10 000 population, 95% confidence interval −0.39 to 0.04, P=0.10; trend change −0.03 prescriptions per 10 000 population per month, −0.06 to 0.003, P=0.07).

### Mometasone

Figure 2 shows the monthly prescribing rates for mometasone in English speaking and French speaking Canadian provinces. As with etanercept, we saw no clinically important or statistically significant change in the level or trend of differences in prescribing rate between English speaking and French speaking provinces (level change −3.61 prescriptions per 10 000 population, −10.51 to 3.29, P=0.30; trend change −0.08 prescriptions per 10 000 population per month, −0.57 to 0.40, P=0.73).

### Tegaserod

In contrast to the first two drugs described, US direct to consumer advertising for tegaserod seemed to have a strong influence on Canadian prescribing. Figure 3 shows the monthly prescribing rates for tegaserod. The February 2003 campaign, which contained no US network news advertising, had no significant impact on prescribing rates and was incorporated into the pre-advertising period. In contrast, a level increase of 0.56 prescriptions per 10 000 population (0.37 to 0.76, P<0.001) in the difference in prescribing rate between English speaking and French speaking provinces occurred immediately after the August 2003 campaign. We found no statistically significant change in trend (−0.003 prescriptions per 10 000 population per month, −0.03 to 0.02, P=0.77). Overall, this represents an estimated 42% increase in the first month after direct to consumer advertising. However, this difference did not persist despite continued advertising throughout the study period. Within two years of direct to consumer advertising, prescribing rates were again virtually identical between English speaking and French speaking regions.

Using the same start date for direct to consumer advertising, we found a similar increase in Medicaid prescription rates of tegaserod. Figure 4 shows that the pre-advertising upward trend in tegaserod use was substantially higher in US Medicaid than in Canada. After national network news direct to consumer advertising, we saw an increase in the level of prescribing in the United States; the number of prescriptions per 10 000 enrolees increased by 5.70 (3.65 to 7.75, P<0.001). As in Canada, we found no statistically significant change in prescribing trends (−0.62 prescriptions per 10 000 enrolees per quarter, −1.52 to 0.27, P=0.15). Overall, the estimated increase in prescribing in the first quarter of direct to consumer advertising was 56% higher than would have been expected and greater than the 42% increase seen in Canada.

**Figure fig4:**
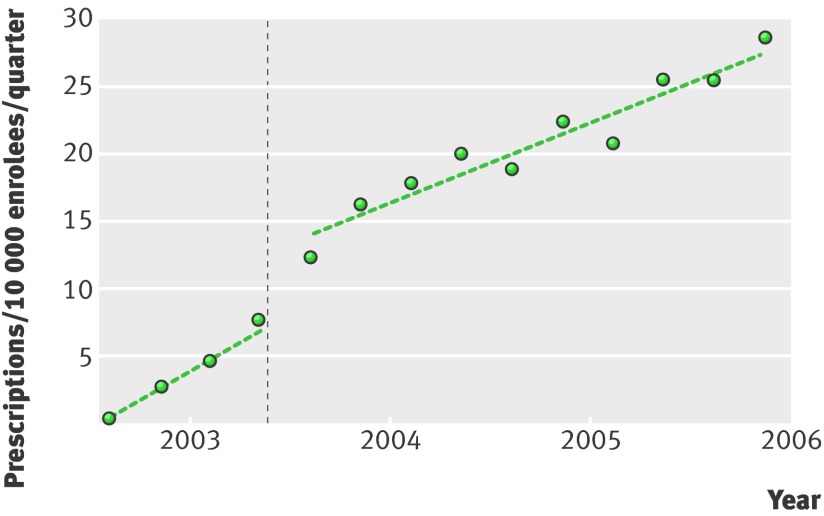
**Fig 4** Number of tegaserod prescriptions per 10 000 enrolees per quarter in US Medicaid programmes. Vertical line indicates start of new TV advertising campaign in third quarter of 2003. Fitted trend line represents fitted interrupted time series analysis for rate of use in Medicaid

## Discussion

During the past decade, drug manufacturers have substantially increased spending on direct to consumer advertising.^1^ To our knowledge, this study is the first analysis that uses a concurrent control group to evaluate the impact of such advertising on use of specific drugs. We found that for two of three drugs the US direct to consumer advertising had no apparent impact on Canadian prescribing rates, and for one drug (tegaserod) we saw a short lived effect. These mixed findings are surprising, as we included several expensive advertising campaigns that were highly recalled by consumers.^27^ ^28^ Our empirical results raise important questions about whether and how prescribing trends for specific drugs respond to advertising directed at consumers. Thus, they have important implications for the ongoing debate about the benefits and harms of direct to consumer advertising.

### Possible explanations

We believe that the differential responses to direct to consumer advertising that we saw may be related to the characteristics of the drugs examined. Although all of the study drugs are primarily used for relieving symptoms, they differ in important ways. For example, etanercept requires referral to a specialist and intravenous administration, making the pathway between direct to consumer advertising and drug use complicated. Thus, the effect of advertising probably differs substantially from that of drugs prescribed predominantly in primary care settings. Furthermore, tegaserod, unlike the other study drugs, was the only drug approved for its indication in Canada.^29^ ^30^ In contrast, the other drugs studied all had competitors within the same drug class. In such markets, direct to consumer advertising might protect against drops in levels of use, rather than expanding use. Other characteristics, such as effectiveness, may also be important. A meta-analysis of short term placebo controlled trials of tegaserod indicates that the number needed to treat for one patient to have some improvement in their gastrointestinal symptoms is about 17, suggesting that most patients trying tegaserod for the first time were unlikely to derive symptomatic benefit.^30^ This may explain, in part, why the changes in use for this drug were short lived.

Our results also suggest that when direct to consumer advertising does increase use, a dose-response relation with the level of exposure to advertising exists. Our results in US Medicaid programmes estimated a larger increase than in Canada, in both absolute and percentage terms. Although the immediate change in use in the United States was larger than in Canada, assessing the comparative long term effect of advertising in the United States is difficult, because no concurrent control group is available. Nevertheless, the observed US Medicaid prescribing rates returned to the pre-direct to consumer advertising trend around the same time as in Canada (mid-2005). Furthermore, use of tegaserod was both higher and growing faster in Medicaid before direct to consumer advertising, suggesting that other factors were driving these differential trends. For example, we cannot rule out between country differences in physician directed marketing activities.^31^

### Strengths and limitations

The major strength of our study is the use of a strong quasi-experimental design with a comparable and concurrent control group. Moreover, our study design controlled for difference in both pre-existing level and trend and explicitly considered the timing of advertising campaigns. This method controls for differences in characteristics between language regions of Canada that remained constant or changed predictably over time, such as culture or patterns of general medical practice. Indeed, other differences such as variation in provincial drug reimbursement plans would bias our results only if they coincidentally changed when the individual direct to consumer advertising campaigns started. We could find no evidence that this occurred for any of the drugs studied. None the less, exclusion from provincial formularies might constrain the effects of successful advertising campaigns. However, most private insurance plans in Canada do not have formularies and cover most of the population.^32^ Moreover, although Ontario and Alberta both excluded tegaserod from their public drug programmes, the effect of direct to consumer advertising was apparent in both provinces (data not shown).

The study has other limitations. Firstly, generalising beyond the three drugs that met our inclusion criteria is difficult. Secondly, we do not have information on whether these drugs were subject to disease awareness advertising by companies that did not mention the brand name. However, this would bias our results only if it was similarly timed, and we found no indication for mometasone or etanercept of increased use coincident with branded direct to consumer advertising, thus making it unlikely. Thirdly, variation in drug coverage, the overall health system, culture, levels of exposure to advertising, or television viewing patterns might result in the effect of direct to consumer advertising differing between drugs and between countries. However, the percentage increase in and duration of effect for tegaserod was similar in both countries. In terms of drug coverage, more than 60% of Canadians are covered by generally unrestrictive employer based private drug plans, and less than 20% of these plans use formularies to limit access to specific drugs.^32^ Although the Canadian and US health systems vary substantially, studies indicate similar access to primary care and willingness of physicians to fulfil patients’ requests for specific prescription drugs in the two countries.^15^ ^33^ Overall, the striking initial effect of direct to consumer advertising on tegaserod prescribing rates provides evidence that exposure to US advertising is sufficient to influence Canadian prescribing.

### Implications

The implications of our analysis are threefold. Firstly, it indicates that illicit cross border exposure to direct to consumer advertising has the potential to modify drug use, even where such advertising is technically prohibited. As advertising over global mediums such as the internet increases, this phenomenon may grow in importance. Secondly, to our knowledge, these results are the strongest evidence that direct to consumer advertising can increase use of a drug that was removed from the market as a result of concerns about safety. Finally, our findings suggest that the impact of direct to consumer advertising campaigns is mixed, as they seem to work for some drugs and not others. If the overall impact of direct to consumer advertising is limited or variable, then a substantial portion of expenditure on such advertising—borne by governments, insurers, and patients in the form of higher costs or by companies as reduced profits—may be better spent elsewhere. Previous commentary may have overemphasised the impact of direct to consumer advertising for many individual drugs for which evidence that it increases use is either weak or non-existent.^2^ Until we better understand how direct to consumer advertising modifies prescribing for particular drugs, debates about its positive and negative consequences will continue to be based on conjecture rather than strong evidence.

**What is already known on this topic**

Although direct to consumer advertising (DTCA) of prescription drugs remains controversial, no controlled studies have investigated its impact on prescribing
In the absence of such evidence, both opponents and proponents of DTCA have generally assumed it to be highly effective at increasing the use of advertised drugs

**What this study adds**

DTCA campaigns seem to have mixed effectiveness; drug use did not increase for two of three drugs studied
Despite prohibitions, DTCA can influence prescribing across national borders
The drug (tegaserod) for which use increased with DTCA was eventually withdrawn owing to safety concerns

## Notes

We are grateful to IMS Health Canada, ESI Canada, and Eloda for providing data for this research. We also thank Alyce Adams, Katherine Swartz, Cory Cowan, Ellen Aquilina, Véronique Laramée, Katy Backes Kozhimannil, and Rebecca Anhang-Price for valuable assistance.

Contributors: MRL participated in the design of the study protocol, data collection, statistical analysis, and drafting and revision of the manuscript. SRM participated in the design of the study protocol, data collection, and drafting and revision of the manuscript. SBS participated in the design of the study protocol and drafting and revision of the manuscript. All authors accept full responsibility for the work and/or the conduct of the study, had access to the data, and controlled the decision to publish. MRL is the guarantor.

Ethical approval: The research was appraised and granted an exemption from review by the Harvard Pilgrim Health Care Human Studies Committee.

Provenance and peer review: Not commissioned; externally peer reviewed.

## Notes

**Cite this as:** *BMJ* 2008;337:a1055
